# Editorial: Invasive alien plant species: From the molecular to the economic approach

**DOI:** 10.3389/fpls.2023.1185567

**Published:** 2023-03-30

**Authors:** Mirjana Ljubojević

**Affiliations:** Faculty of Agriculture, University of Novi Sad, Novi Sad, Serbia

**Keywords:** allelochemicals, neolitization, nitrogen forms, physiology, plant invasiveness

Invasive alien species (IAS) are plants, animals, pathogens and other organisms that are not natural in a certain ecosystem and that can cause economic or environmental damage or adversely affect human health (Ljubojević et al., 2022). Plant invasiveness is a globally recognized environmental and economic problem (Bang et al., 2022). Today, the loss of biodiversity occurs due to intensive anthropogenic activities, like over-exploitation of species, urbanization and industrialization, environmental pollution, land-use shift, the introduction of IAS as well as climate change (Ren and Duan, 2017). IAS introduction has many advantages and disadvantages, leading to numerous ecosystem services and disservices.

Fostered by climate changes (Kariyawasam et al., 2019) and inherited (inner) ability to adapt to various conditions, IAS treat to suppress natural vegetation. Theoharides and Dukes (2007) defined four main stages of invasion: introduction, naturalization, colonization, and dispersal. Milanović et al. (2020) stated that alien (especially invasive) plant species differ from native species in different morphological characteristics such as specific leaf area, height, seed size or flowering duration, where invasive species showed significant dominance in the investigated characteristics. A list of invasion-promoting traits is being amended as novel research is being conducted, from seed characteristics (Ljubojević et al., 2021) to the whole-plant level (Bajwa et al., 2016). With such high adaptive potential alien species develop traits that allow them to successfully cope with the changes in climate or habitats (Dukes and Mooney, 1999). However, being very adaptive, fast-growing, and not infrequently highly ornamental, those species provide numerous ecosystem services. If viewed exclusively as ‘weeds’ then many resources would be invested in the eradication measures of invasive alien species, with little certainty about the desired outcome. Thus, this Research Topic gathered recent findings from around the world, providing insight into differences in gene expression, morphology, physiology and resource utilization by invasive alien species, that provided them with successful habitat invasion.

As shown by (Nunes et al.) weedy invaders spread rapidly due to successful adaptation and naturalization, affecting both natural and agricultural ecosystems. One of the most invasive weed species in the sub-tropical and continental climate is *Arundo donax* L. from *Poaceae* family. Owing to the chemical and histological modifications, this species successfully inhabit the Mediterranean basin. Another invasion pathway includes soil usage and nutrient acquisition. Two papers in this collection investigated the effects of nitrogen forms on invasiveness capability in *Xantium* species. The invasive plant *Xanthium strumarium* L. prefers nitrate relative to ammonium, and mainly invades nitrate-dominated environments, while its co-occurring native congener *X. sibiricum* Widd. prefers ammonium (Zhang et al.), but the molecular mechanism underlying these processes was unknown. All tested transcripts were significantly up-regulated by GA1 and GA4 in *X. sibiricum*. XsiGA3OX1a, which was also induced by ammonium, may be involved in this regulation (Zhang et al.).

However, our knowledge of the mechanisms involved remains limited. Another weedy, annual or perennial species *Pedicularis kansuensis* Maxim. responses differently to the nitrogen forms and availability. Investigating the second largest grassland in China – Bayinbuluk Grassland (as a part of Bayinbuluk Grassland Ecosystem Research Station, Chinese Academy of Sciences) Liu et al. provided new empirical evidence that the successful benign invasion of annual *P. kansuensis* (non-transformer invasive species) can be associated with the reduction of native species coverage and photocompetition. This study showed that nitrogen enrichment can effectively inhibit *P. kansuensis* invasion due to the increased photocompetence of the native species.

Existing greenery and its potential green products should be viewed as a source of capital that we have borrowed from nature and which we should leave to our descendants as an inheritance, instead of wasting it by inappropriate maintenance. Research covering the utilization of IAS in innovation opportunities – nature-based solutions (biopesticides, biofuels and similar) - is more than needed. In general, biopesticides for plant disease management include the exploitation of natural organisms, and derived products, biocontrol formulations, essential oils, botanical extracts as well as nano biopesticides (Meshram et al., 2022). Allelochemicals as plant secondary metabolites, released into the environment *via* root exudation, leaching by precipitation, volatilization, or decomposition of plant tissues have the potential as agricultural biopesticides (Scavo and Mauromicale, 2021). Allelochemicals are considered techniques for increasing yields and quality through sustainable disease, pest and weed management. A review paper by Rahaman et al. provided an overview regarding the allelopathy and allelochemical types, investigating techniques, modes of action, production pathways, factors influencing the production of allelochemicals in plants, genetical manipulation as well as the significance of rice allelopathy in sustainable agriculture. Presented research concluded that rice allelopathy is one of the best options for environmentally friendly weed management in rice.

To prevent, manage and control invasive plant species, one of the most used tools are species distribution models. The output of these models can foster knowledge regarding population characteristics, spatial abundance patterns or species performance (Silva et al.). Although ecological theory suggests a direct link between fitness and suitability, this link is often absent, due to multiple reasons. Investigating the invasiveness of *Acacia* species in Chile, Silva et al. did not observe an association between environmental suitability and tree growth thus plans to control invasive species should be cautious when assuming this unequivocal relationship.

Research studies employing basic quantitative analyses such as morphological, anatomical, physiological, biochemical and molecular, combined with constant monitoring and qualitative data acquisition, lead to a further understanding of invasion mechanisms. However, combat with invaders tends to be a failure if observed only as a treat. Some benign invasions can be regarded as biodiversity amendment as long as natural vegetation is not disturbed, providing novel ecosystem services that can be beneficial for both nature and humans. Complete understanding of invasive capability and habitat occupation is interrupted due to a large number of invasive plant species, their life cycle differences, soil and nutrient preferences, adaptive strategies and modifications.

Thus, continuous research and a non-biased approach by all actors - scientists, land managers, policy-makers and the general public must be prioritized to achieve set millennium goals and provide the buffer for the climate-soil-anthropocene-related changes.

## Author contributions

The author confirms being the sole contributor of this work and has approved it for publication.
